# First-line treatment of driver gene-negative metastatic lung adenocarcinoma with malignant pleural effusion: Should chemotherapy be combined with an immune checkpoint inhibitor or bevacizumab?

**DOI:** 10.1007/s10637-024-01424-4

**Published:** 2024-02-22

**Authors:** Yuanyuan Zhao, Ting Mei, Feifei Na, Xiaoman Tian, Rui Ao, Xiangyu Long, Qiang Luo, Ping Duan, Jiang Zhu, Yongsheng Wang, Meijuan Huang, Yongmei Liu, Youling Gong

**Affiliations:** 1 Division of Thoracic Tumor Multidisciplinary Treatment, Cancer Center and State Key Laboratory of Biotherapy, West China Hospital, Sichuan University, Chengdu, 610041 People’s Republic of China; 2 Department of Oncology, Chengdu Pidu District Hospital of Traditional Chinese Medicine, Chengdu, 611730 People’s Republic of China; 3 Lung Cancer Center, West China Tianfu Hospital, Sichuan University, Chengdu, 610213 People’s Republic of China; 4 Department of Oncology, Chengdu Jinniu District People’s Hospital, Chengdu, 610031 People’s Republic of China; 5 Department of Oncology, Sichuan Academy of Medical Sciences and Sichuan People’s Hospital, Chengdu, 610072 People’s Republic of China; 6 Department of Oncology, Sichuan Provincial Guang’An People’s Hospital, Guang’An, 638500, People’s Republic of China; 7 Department of Oncology, Chengdu Xinjin District Hospital of Traditional Chinese Medicine, Chengdu, 611430 People’s Republic of China; 8 Department of Oncology, Chengdu First People’s Hospital, Chengdu, 610095 People’s Republic of China; 9 Department of Oncology, West China Shangjin Hospital, Sichuan University, Chengdu, 611730 People’s Republic of China

**Keywords:** Platinum-based doublet chemotherapy, Antiangiogenic agent, Immune checkpoint inhibitors, Combined treatment, PD-L1 expression

## Abstract

Patients with metastatic lung adenocarcinoma (MLA) and malignant pleural effusion (MPE) without driver gene mutations have a poor prognosis. None of the standard treatment strategies is recommended for such patients. We retrospectively analyzed the efficacy of the first-line treatment for this specific population: standard platinum-based doublet chemotherapy (CT), CT plus an immune checkpoint inhibitor (CT plus ICI), and CT plus bevacizumab (CT plus Bev). A total of 323 eligible patients were enrolled: CT alone (*n* = 166), CT plus Bev (*n* = 72), and CT plus ICI (*n* = 85). Treatment efficacy assessments were performed every two cycles according to the RECIST guidelines. The endpoints were overall survival (OS) and progression-free survival (PFS). Kaplan-Meier (K‒M) curves and the log-rank test were used to compare OS and PFS. *p* < 0.05 was the threshold of significance (statistical software: SPSS). The median follow-up was 11.4 months (range, 2.1–49.6 months). PFS and OS in the CT plus ICI/CT plus Bev cohort were significantly longer than those in the CT group (PFS: 7.8/6.4/3.9 months, *p* < 0.0001; OS: 16.4/15.6/9.6 months, *p* < 0.0001, respectively). CT plus Bev had better PFS and OS than CT plus ICI/CT in PD-L1 < 1% patients (PFS: 8.4/5.0/3.8 months, *p* < 0.0001; OS: 15.6/12.9/9.3 months, *p* < 0.0001). Among patients with PD-L1 1–49%, CT plus ICI led to a longer PFS and OS (PFS: 8.9/5.8/4.2 months, *p* = 0.009; OS: 24.2/18.8/11.5 months, *p* = 0.03). In the cohort with PD-L1 ≥ 50%, CT plus ICI was still the best first-line treatment (PFS: 19.7/13.8/9.6 months, *p* = 0.033; OS: 27.2/19.6/14.9 months, *p* = 0.047). In driver gene-negative MLA with MPE, CT plus Bev or ICI better controlled MPE and significantly prolonged survival compared to CT alone. PD-L1 expression (negative/positive) may be a key factor influencing the choice of CT plus Bev or ICI.

**Supplementary Information:**

The online version contains supplementary material available at 10.1007/s10637-024-01424-4.

## Background

According to the Global Cancer Statistics 2020 report published by the International Agency for Research on Cancer, lung cancer ranks in the top two in incidence and mortality rates [1]. Approximately 80% of lung cancer cases are initially diagnosed at an advanced stage, at which point the incidence of MPE is approximately 10–30%, resulting in a poor prognosis [2–6]. Adenocarcinoma is the most common pathological type of MPE [4]. With the development of antiangiogenic agents and immunological drugs, the preferred first-line systematic treatment is no longer CT alone.

In recent years, Bev has been demonstrated the ability to be able to effectively control MPE either in isolation or in tandem [7–9]. This may stem from the fact that the development of MPE depends on the invasion of tumorigenic pleural cells and elevated levels of vascular endothelial growth factor (VEGF) expression [10, 11]. A phase II clinical trial suggested that an elevated level of VEGF may be linked to a poor prognosis of MPE, and patients who had high VEGF in their MPE demonstrated shorter pleural progression-free survival (PPFS) and OS [9].

Since Bev has not succeeded in prolonging the survival rate within this specific population, the evidence supporting the use of Bev for MPE management remains weak [12, 13]. When the survival of MLA after immunotherapy has been significantly improved, ICIs are recommended as a new first-line option for driver gene-negative MLA based on a combination of CT [6, 14–16], including in patients with MPE [17, 18]. To date, only one study has explored the best first-line treatment for nonsquamous non-small-cell lung cancer (NSCLC) combined with MPE: ICI combined with or without CT [16]. This analysis from Japan showed that CT plus ICI was more effective than ICI alone, even when PD-L1 was highly expressed. However, the researchers studied mainly ICI-based regimens. In the present study, our objective was to compare the following strategies for this specific population: CT, CT plus ICI, and CT plus Bev.

## Patients and methods

Data from 3458 consecutive NSCLC patients treated from February 2017 to October 2022 at West China Hospital, Sichuan University and other participating hospitals were analyzed retrospectively.

### Criteria for inclusion and exclusion

#### Inclusion criteria

The inclusion criteria are as follows: (1) IV NSCLC without treatment, (2) first-line platinum-based CT with or without anti-angiogenesis therapy/ICIs, (3) malignant neoplastic cells identified within the pleural effusion or with a favorable pleural biopsy, (4) Eastern Cooperative Oncology Group (ECOG) performance status (PS) of 0–1, and (5) a projected length of survival exceeding 2 months.

#### Exclusion criteria

The exclusion criteria are as follows: (1) having undergone lung surgery, (2) exhibiting positive driver gene mutation status, (3) having undergone first-line immunotherapy or antiangiogenic treatment, (4) contraindications for the use of drugs such as high risk of bleeding, hypertension crisis, or hypertensive encephalopathy, (5) reversible posterior leukoencephalopathy syndrome, and (6) nephrotic syndrome.

### Data synthesis and statistical methods

#### Data sources

The prescription and other medical data were extracted from the hospital information system (HIS) of each participating hospital.

This retrospective study was approved by the Ethics Review Committee of West China Hospital, Sichuan University, and which waived informed consent.

#### Treatment protocol

Three hundred twenty-three patients diagnosed with primary MLA combined with MPE without driver gene mutations were enrolled in the study. All patients received standard platinum-containing two-drug CT in combination with ICI or Bev. They were categorized into three groups: CT, CT plus ICI, and CT plus Bev. Treatment regimens and doses were chosen based on National Comprehensive Cancer Network (NCCN) guidelines and government approval for the Peoples’ Republic of China [18]: carboplatin or cisplatin combined with pemetrexed or paclitaxel or albumin-bound paclitaxel, Bev and ICIs: pembrolizumab, atezolizumab, camrelizumab, nivolumab, sintilimab, tislelizumab, etc.

#### Study variables

The variables analyzed in the present study were age, sex, performance status (PS) score, smoking, T and N stage, liver/brain metastasis, and treatment strategies (CT, CT plus ICI, and CT plus Bev).

#### Evaluation of efficacy

Treatment efficacy was assessed every two cycles according to the Response Evaluation Criteria in Solid Tumors (RECIST). Evaluation control of pleural effusion was determined by ultrasound of the thorax as described [7, 19, 20]: complete response (CR)—the accumulated effusion disappeared and remained stable for at least 4 weeks; partial remission (PR)—the accumulated effusion fell by 50%, symptoms improved, fluid accumulation did not increase, and fluid remained stable for at least 4 weeks; insignificant relief (NC)—less than 50% of pleural effusion disappeared or symptoms did not significantly change; and progressive disease (PD)—cumulative fluid accumulation increased and symptoms deteriorated.

#### Endpoint definition

PFS was defined as the time between CT initiation and death or disease progression, while OS was defined as the time between treatment initiation and death or the most recent follow-up. OS and PFS were the primary and secondary endpoints for this study, respectively. All cases were followed up through hospitalization, outpatient service, or telephone until the death of the patient or the end of follow-up in October 2022.

#### Statistical methods

Baseline characteristics were compared between groups via the chi-square test. K‒M curves and the log-rank test were used to compare PFS and OS between groups. Differences in pleural effusion control between different treatment strategies were assessed using logistic regression analysis. Forest plots were drawn for subgroup analysis. All *p*-values were two-sided and were considered significant at *p* < 0.05.

## Results

### Patients’ characteristics

Patient characteristics are summarized in Table 1. Patients were categorized into three cohorts: CT alone (*n* = 166), CT combined with Bev (*n* = 72), and CT combined with ICI (*n* = 85). Among these patients, the median age was 58 years, with a range of 28–72 years. Most of the patients were males (233/323, 72.1%), with a PS = 1 (320/323, 99.1%). There were 47 (14.6%) and 57 (17.6%) patients with liver/brain metastasis, respectively.

**Table 1:** Baseline characteristics of patients with advanced lung adenocarcinoma with MPE without driver gene mutation

Variable	CT plus ICI (*n* = 85)	CT plus Bev (*n* = 72)	CT (*n* = 166)	*p-*value
Age, years				
< 60	33(38.8)	31 (43.1)	70 (42.2)	0.874
≥ 60	52(61.2)	41 (56.9)	96 (57.8)	
Sex				
Male	63 (74.1)	49 (68.1)	121 (72.9)	0.589
Female	22 (25.9)	23 (31.9)	45 (27.1)	
PS score				
0	2 (2.4)	1 (1.2)	0 (0.0)	0.166
1	83 (97.6)	71 (98.8)	166 (100)	
Smoking				
Yes	50 (58.8)	45 (62.5)	99 (59.6)	0.885
No	35 (41.2)	27 (37.5)	67 (40.4)	
T stage				
T1-2	13 (0.0)	19 (6.0)	36 (3.0)	0.227
T3-4	72 (18.8)	53 (19.3)	130 (17.9)	
N stage				
N0-1	15 (17.6)	12 (18.1)	24 (10.4)	0.785
N2-3	70 (82.4)	60 (83.3)	142 (85.5)	
Liver metastases				
Yes	12 (14.1)	10 (13.9)	25 (15.1)	0.964
No	73 (85.9)	62 (86.1)	141 (84.9)	
Brain metastases				
Yes	14 (16.5)	14 (19.4)	29 (17.5)	0.885
No	71 (83.5)	58 (80.6)	137 (82.5)	

*CT plus ICI* chemotherapy plus an immune checkpoint inhibitor, *CT plus Bev* chemotherapy plus bevacizumab, *CT* chemotherapy

### Local-control rate (LCR) of MPE and the impact on survival

Pairwise comparisons were made by logistic regression analysis in different treatment groups for LCR of MPE. Both CT plus Bev and CT plus ICI were significantly better than CT in terms of the LCR of MPE (both *p* < 0.001), and the CT plus Bev seemed to be the optimal one (HR, 1.688; 95%CI = 1.096–3.182; *p* = 0.043). Depending on PD-L1 expression, CT plus Bev had better LCR of MPE than CT plus ICI/CT in PD-L1 < 1% patients (HR, 2.647/12.708; *p* = 0.015/ < 0.001, respectively), while CT plus ICI performed better than CT plus Bev/CT in PD-L1 ≥ 50% (*p* = 0.039/0.030, respectively). A higher LCR of MPE significantly prolonged survival (PFS, 11.8 vs. 3.6 months; OS, 21.1 vs. 9.8 months; both *p* < 0.0001) according to K‒M analysis (Fig. S1).

### Impact of different treatment regimens on PFS

**Fig. 1:**
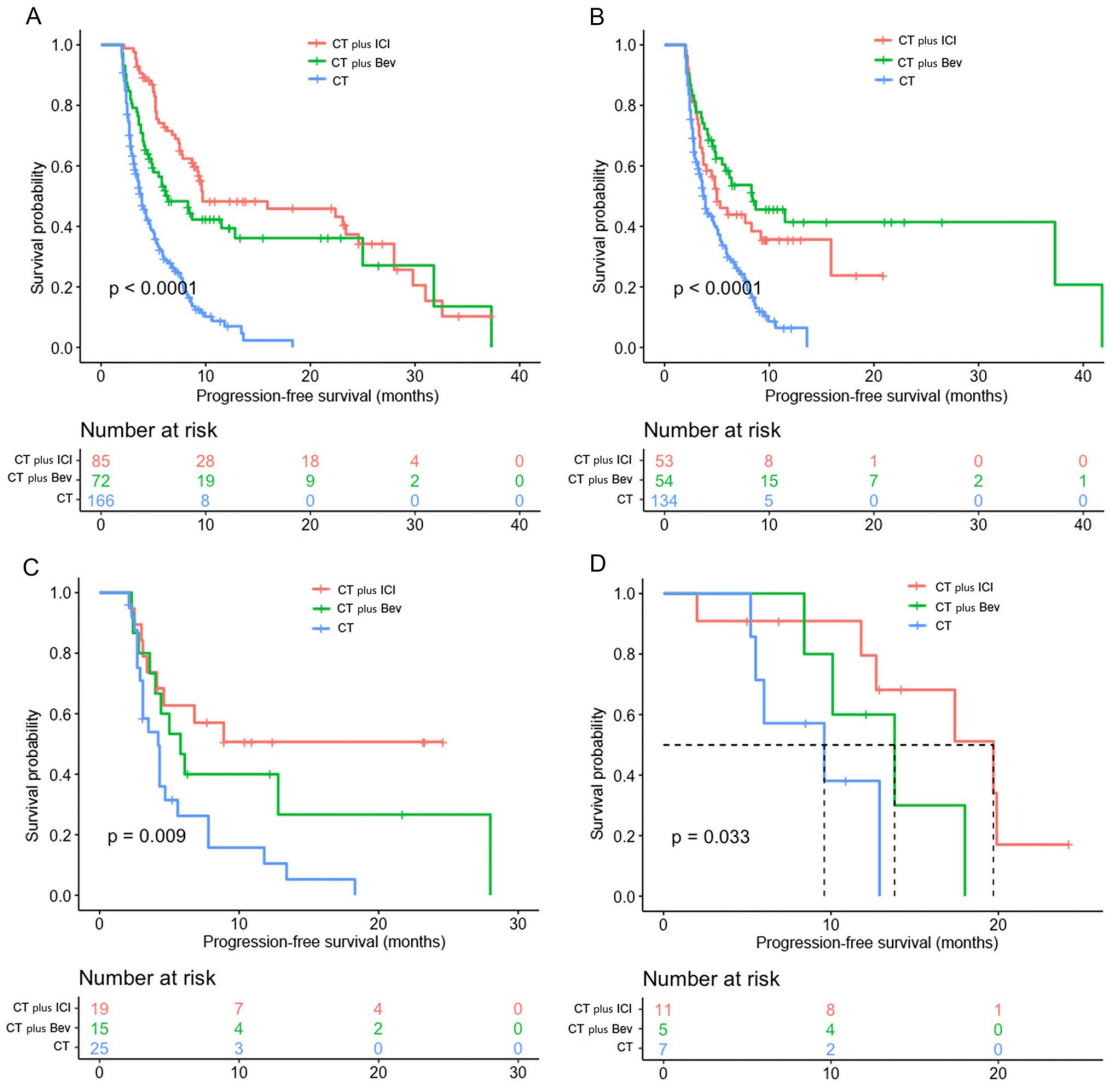
Kaplan-Meier curves for PFS of three different treatment strategies. **A** Kaplan-Meier curves of PFS for the different treatment strategies in all patients; **B** Kaplan-Meier curves of PFS for the different treatment strategies in patients with PD-L1 levels < 1%; **C** Kaplan-Meier curves of PFS for the different treatment strategies in patients with PD-L1 levels of 1–49%; **D** Kaplan-Meier curves of PFS for the different treatment strategies in patients with PD-L1 levels ≥ 50%

The combined treatment group outperformed the CT-only group in terms of PFS (7.8/6.4/3.9 m, *p* < 0.0001) (Fig. 1A). In PD-L1 < 1% of patients, CT plus Bev provided a longer PFS than CT plus ICI and CT (8.4/5.0/3.8 m, *p* < 0.0001) (Fig. 1B), while CT plus ICI performed better in patients with PD-L1 positivity (PD-L1 = 1–49%: 8.9/5.8/4.2 m, *p* = 0.009; PD-L1 ≥ 50%: 19.7/13.8/9.6 m, *p* = 0.033) (Fig. 1C, D).

### Impact of different treatment regimens on OS

**Fig. 2:**
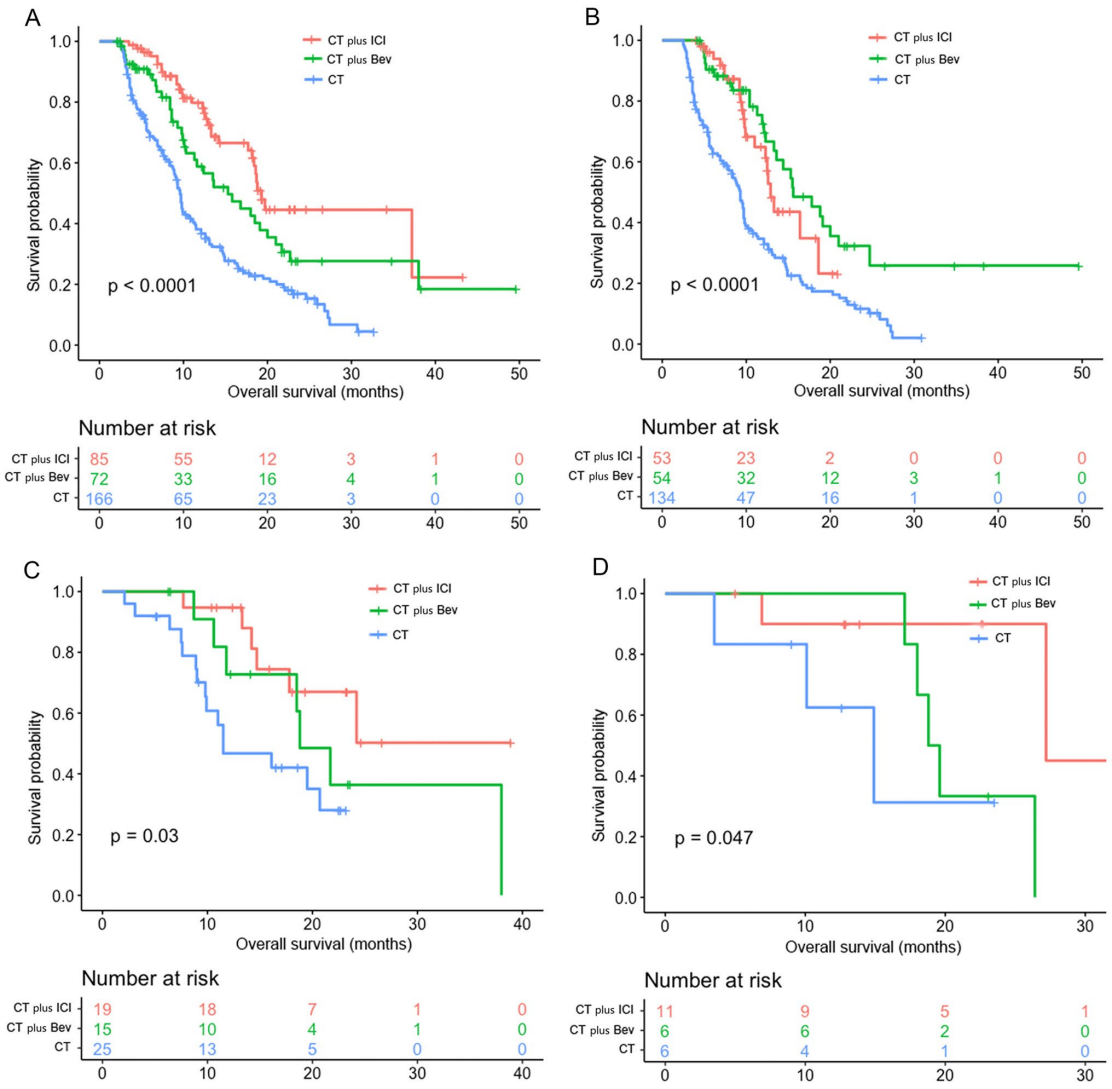
Kaplan-Meier curves for OS of three different treatment strategies. **A** Kaplan-Meier curves of OS for the different treatment strategies in all patients; **B** Kaplan-Meier curves of OS for the different treatment strategies in patients with PD-L1 levels < 1%; **C** Kaplan-Meier curves of OS for the different treatment strategies in patients with PD-L1 levels of 1–49%; **D** Kaplan-Meier curves of OS for the different treatment strategies in patients with PD-L1 levels ≥ 50%

The OS in the CT plus ICI/CT plus Bev group was significantly longer than that in the CT group (16.4/15.6/9.6 months, *p* < 0.0001) (Fig. 2A). The results of OS were similar to those of the PFS in different PD-L1 expression groups: for PD-L1 < 1%, the CT plus Bev group exhibited a longer OS (15.6/12.9/9.3 m, *p* < 0.0001) (Fig. 2B). The CT plus ICI group had better OS than the CT plus ICI/CT groups among patients with PD-L1 = 1–49% (24.2/18.8/11.5 m, *p* = 0.03) (Fig. 2C) and PD-L1 ≥ 50% (27.2/19.6/14.9 m, *p* = 0.047) (Fig. 2D).

### Univariate and multivariable analysis

**Fig. 3:**
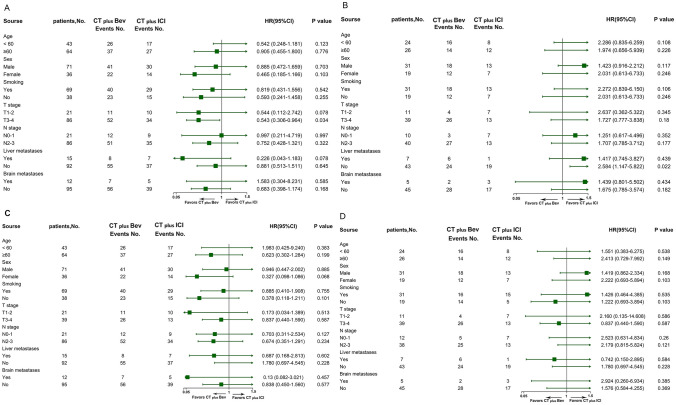
Forest plot of PFS and OS in combined treatment group patients with PD-L1 negative/positive expression. **A** Forest plot of PFS for the different treatment strategies in patients with PD-L1 negative expression; **B** Forest plot of PFS for the different treatment strategies in patients with PD-L1 positive expression; **C** Forest plot of OS for the different treatment strategies in patients with PD-L1 negative expression; **D** Forest plot of OS for the different treatment strategies in patients with PD-L1 positive expression. The point estimate of HR = 1 was used as the futility line, the left side of the futility line was the CT plus Bev group, and the right side of the futility line was the CT plus ICI group. When the 95% CI of HR included 1, that is, when the horizontal line segment in the forest plot intersected the futility line, it indicated that the difference in survival between the two groups was not statistically significant. When the horizontal line segment did not intersect with the futility line and was to the right of the futility line, it meant that the survival of CT plus ICI group was better

PFS and OS in PD-L1-negative and PD-L1-positive patients were analyzed separately within the combination therapy group (Fig. 3A, B for PFS and C, D for OS). Survival rates (age, sex, smoking status, T stage, N stage, liver metastases, and brain metastases) were compared between different subgroups of the combination groups. Forest plots showed that patients with T stage 3–4 in the CT plus Bev subgroup had longer PFS when PD-L1 expression was negative (HR, 0.543; *p* = 0.034) (Fig. 3A). There was no significant difference in survival between the other subgroups.

Univariate analysis of patients revealed that CT plus ICI/Bev was associated with longer PFS (hazard ratio (HR) = 0.266, 95%CI = 0.187–0.377/HR = 0.401, 95%CI = 0.282–0.571, both *p* < 0.001) and OS (HR = 0.337, 95%CI = 0.227–0.499/HR = 0.545, 95%CI = 0.374–0.793, both *p* < 0.001) than CT group, multivariate analysis also revealed that combination regimens were independent predictors of survival prognosis (*p* < 0.05). In addition, the male sex, smoking status, brain metastases status, and T stage 3–4 subgroups were associated with superior OS, among which smoking and T stage 3–4 were found to be independent prognostic factors for OS (*p* = 0.020/0.021, respectively) (Table 2).

**Table 2:** Univariate and multivariate analysis of PFS and OS in present study (*n* = 323)

Variable	Univariate cox regression		Multivariate cox regression	
	HR (95% CI)	*p*-value	HR (95% CI)	*p*-value
**Progression-free survival**				
Age, years				
≥ 60 vs. < 60	0.772(0.595–1.003)	0.053		
Sex				
Male vs. female	1.218(0.920–1.613)	0.169		
Smoking				
Yes vs. no	0.999(0.766–1.303)	0.996		
Liver metastasis				
Yes vs. no	1.282 (0.897–1.832)	0.173		
Brain metastasis				
Yes vs. no	1.276(0.922–1.766)	0.141		
PS score				
2–3 vs. 0–1	5.318(0.743–38.086)	0.096		
T stage				
3–4 vs. 1–2	1.193(0.860–1.654)	0.291		
N stage				
2–3 vs. 0–1	1.314(0.921–1.876)	0.132		
Treatment				
CT plus ICI vs. CT	0.266 (0.187–0.377)	< 0.001	0.266 (0.187–0.377)	< 0.001
CT plus Bev vs. CT	0.401 (0.282–0.571)	< 0.001	0.401 (0.282–0.571)	< 0.001
CT plus ICI vs. CT plus Bev	1.510 (1.000–2.281)	0.050	1.510 (1.000–2.281)	0.050
**Overall survival**				
Age, years				
≥ 60 vs. < 60	1.144(0.863–1.515)	0.350		
Sex				
Male vs. female	1.525(1.119–2.078)	0.008	0.793(0.410–1.533)	0.793
Smoking				
Yes vs. no	1.583(1.177–2.128)	0.02	2.105(1.123–3.943)	0.020
Liver metastasis				
Yes vs. no	1.140 (0.776–1.675)	0.504		
Brain metastasis				
Yes vs. no	1.472(1.064–2.037)	0.020	1.168(0.834–1.636)	0.366
PS score				
2–3 vs. 0–1	5.038(0.701–36.219)	0.108		
T stage				
2–3 vs. 0–1	1.298(0.907–1.857)	0.154		
N stage				
3–4 vs. 1–2	1.761(1.173–2.643)	0.006	1.632(1.075–2.477)	0.021
Treatment				
CT plus ICI vs. CT	0.337 (0.227–0.499)	< 0.001	0.313(0.209–0.470)	< 0.001
CT plus Bev vs. CT	0.545 (0.374–0.793)	< 0.001	0.627(0.421–0.934)	0.022
CT plus Bev vs. CT plus ICI	1.617 (1.010–2.621)	0.049	1.002(0.672–3.283)	0.076

## Discussion

Patients first diagnosed MLA with MPE has a poor likelihood of survival and short life expectancy, especially among those without any driver gene mutations. For the initial treatment of these patients, the NCCN guidelines recommend platinum-based double-agent CT in combination with either ICI or Bev [18]. For the first time, the present data show that combination therapy was superior to CT alone in terms of both survival time (PFS, 7.8/6.4/3.9 months; *p* < 0.0001; OS, 16.4/15.6/9.6 months; *p* < 0.0001) and the LCR of MPE (both *p* < 0.001). Whether combining CT with ICI or Bev is more advantageous depends on PD-L1 expression status (positive/negative).

For metastatic lung cancer patients, previous systemic treatment regimens did not achieve satisfactory survival benefits until the advent of the immunization era [21]. Patients diagnosed with MPE who receive immunotherapy exhibit a longer median OS than non-ICI cohorts [22], while combination therapy with ICI has a survival benefit over ICI alone [16]. According to Bahil Ghanim et al. [22], immunotherapy has the potential to achieve a longer OS than no ICI. Their data encompass not only lung cancer (69/56.1%) but also other malignancies, such as mesothelioma and breast cancer. A total of 100 patients (83.3%) declined immunotherapy, which may have biased their results. A retrospective multicenter cohort study conducted by Hayato Kawachi et al. [16] based on immunotherapy indicated that for the PD-L1-high cohort, the CT plus ICI group exhibited a significantly higher objective response rate (ORR) and disease control rate (DCR) than the ICI-alone group (76.7% vs. 34.6%, *p* = 0. 0015; 93.3% vs. 50.0%, *p* = 0.0003, respectively). In addition, in the CT plus ICI cohort, the data revealed no significant difference in ORR (79.0% vs. 50.0%, *p* = 0.0653) or DCR (89.5% vs. 88.9%, *p* = 0.9543) between the BEV and non-BEV groups. Our findings are partially consistent with these findings: CT plus ICI provided a greater survival benefit in PD-L1-positive patients (*p* < 0.05). CT plus ICI therapy was the treatment strategy of choice for the PD-L1-high cohort (PFS, 19.7/13.8/9.6 months; *p* = 0.033; OS, 27.2/19.6/14.9 months; *p* = 0.047, respectively). Based on the data presented above, our study analyzed in greater depth the standard platinum-containing CT and CT plus Bev treatment modalities. Regardless of PD-L1 expression, CT significantly reduced both PFS and OS. The CT plus Bev strategy was best among the three modalities when the tumor was PD-L1-negative (PFS, 8.4/5.0/3.8 months; *p* < 0.0001; OS, 15.6/12.9/9.3 months; *p* < 0.0001, respectively). In the IMpower 131 study, which compared the PD-L1-negative subgroup of atezolizumab plus (carboplatin plus nab-paclitaxel) Cnp vs. the Cnp group, neither PFS nor OS benefited if the patients were diagnosed with TC0 or IC0 (mPFS, 5.7 vs. 5.6 months; HR = 0.82; mOS, 14.0 vs. 12.5 m; HR = 0.87) [23]. Although the mPFS was better in the PD-L1-negative group of IMpower 130 than in the atezolizumab plus CT group (6.2 vs. 4.7 months, HR = 0.72), the mOS was not absolutely better (15.2 vs. 12.0 months, HR = 0.81) [24]. No absolute survival benefit was shown between the CT plus ICI and CT plus placebo cohorts. The results of the Bev study (BEYOND study) in China, consistent with the E4599 study, indicated that the combination treatment of these two agents offered significant survival benefits for driver gene-negative MLA patients, with survival data that were not inferior to published data from CT plus ICI trials [25, 26]. Internationally, some scholars have also focused on the question of whether CT plus ICI therapy should be prioritized for PD-L1-negative patients. Their conclusions varied and were derived from retrospective network meta-analyses. According to a meta-analysis of 14 clinical trials (CT plus Bev or CT plus ICI), the improvement in PFS associated with CT plus Bev/ICI was not significant in the PD-L1 TPS < 1% subgroup (*p* = 0.56) [27]. Pembrolizumab combined with CT showed a better benefit for OS and PFS than other therapies (CT, CT plus ICI, monoimmunotherapy, and doublet immunotherapy) [28, 29], and the data from Jiaqi Li et al. showed that CT plus Bev ranked second behind the combination of nivolumab/Bev/CT in terms of PFS [30]. Prospective studies should be performed to answer this question.

The four-drug combination regimens appear to provide a survival benefit to patients over the three-drug regimens [30]. The IMpower150 study is the first phase III clinical study of CT plus ICI plus Bev in stage IV nonsquamous NSCLC, and it showed a statistically significant improvement in PFS and OS compared to the three-drug modality (either CT combined with immunotherapy or anti-angiogenesis) [31]. In addition, the ONO-4538-52/TASUKI-52 study [32] indicated that the PFS in the carboplatin and paclitaxel plus Bev plus Nivolumab group was prolonged compared to that in the carboplatin and paclitaxel plus Bev plus placebo group (12.1 vs. 8.1 months, HR = 0.56). It seems that the four-drug regimen might be more beneficial for MLA patients with MPE. However, considering the economics and insurance policies in China, the high financial burden of combination drugs led very few patients to choose the four-drug regimen in the present study. Therefore, more studies are needed for this particular population.

Although systemic therapies have shown relatively satisfactory outcome assessments, managing recurrent and obstinate MPE necessitates the administration of topical treatment. In addition to the commonly employed clinical thoracic perfusion therapy involving agents such as platinum and interleukin-2, Bev is also being used progressively for intrathoracic infusion therapy in MPE patients. Treatment with Bev alone or in combination, administered intrapleurally for MPE, resulted in a good overall response rate and better quality of life [7, 13, 33–36]. Concurrently, ambulatory small catheter drainage and pleurodesis are both considered feasible surgical options [37, 38]. Lobectomy or sublobectomy with pleurodesis utilizing video-assisted thoracoscopic surgery (VATS) has been found to enhance survival [39]. We did not undertake an extensive evaluation of localized intrathoracic treatments because it is difficult to include too many therapeutic factors in real-world studies to assess their efficacy. In patients with MPE, prompt administration of systemic therapy after adequate drainage remains the standard of care, adding localized intrathoracic therapy should be administered only when systemic therapy does not adequately control the pleural fluid.

Several limitations of this study are noteworthy. First, as a retrospective clinical study, it inevitably suffered from selectivity bias and information bias. PD-L1 assays and reagents vary between hospitals, possibly leading to bias in the baseline data. Second, the sample size of this study was relatively small. We included MLA with an initial diagnosis of wild-type combined with MPE, and few of these patients were diagnosed early enough and treated regularly in the clinic, making it difficult to identify and enroll such patients and analyze them, but this is the first study to date to compare head-to-head efficacy in these patients. In addition, unlike the IMpower clinical trials and some retrospective studies of ICI for advanced NSCLC [28, 30], we were not able to collect or analyze the incidence of toxicity; this deficiency may have compromised the completeness of the follow-up of the enrolled patients. Finally, due to the small sample size, the differences in efficacy between different CT regimens and immunization regimens were not further analyzed.

In conclusion, the combination of CT with ICI/Bev provided better control over MPE than CT alone and was also linked to markedly extended survival in driver gene-deficient MLA patients. The expression of PD-L1 might be a decisive factor in the choice of treatment CT combined with ICI/Bev. In addition, the male status, smoking status, brain metastases status, and T stage 3–4 subgroups were associated with performed longer OS, and smoking status and T stage 3–4 were found to be independent prognostic factors for OS. Prospective studies are needed to confirm these findings.

### Supplementary Information

Below is the link to the electronic supplementary material.

Supplementary file1 (TIF 407 KB)

## Data Availability

The data that support the findings of this study are available from the corresponding author upon reasonable request.
