# Supplementary figures and images for: First-line treatment of driver gene-negative metastatic lung adenocarcinoma with malignant pleural effusion: Should chemotherapy be combined with an immune checkpoint inhibitor or bevacizumab?
Source: Invest New Drugs. 2024 Feb 22;42(2):196–206. doi: 10.1007/s10637-024-01424-4 (PMC10944392; doi:10.1007/s10637-024-01424-4)

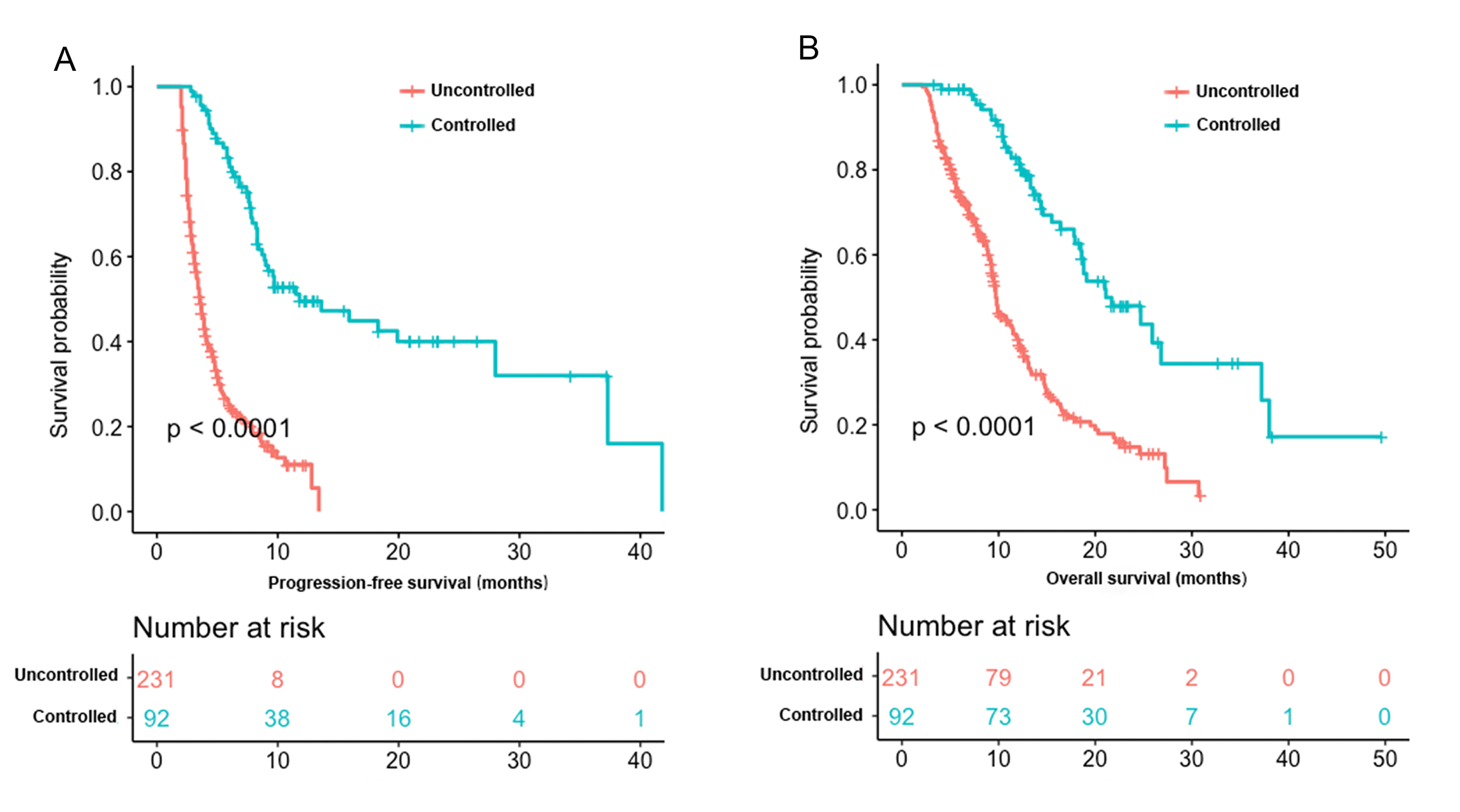

Supplement: Supplementary file 1 — Supplementary file1 (TIF 407 KB) [file 10637_2024_1424_MOESM1_ESM.tif]
